# Supplementary material for: Identification of MYEOV-Associated Gene Network as a Potential Therapeutic Target in Pancreatic Cancer
Source: Cancers (Basel). 2022 Nov 4;14(21):5439. doi: 10.3390/cancers14215439 (PMC9655857; doi:10.3390/cancers14215439)
Supplement: Supplementary file 1 [file cancers-14-05439-s001.zip › cancers-1996998-supplementary.pdf]

Supplementary materials

Table S1. Primers used in this study.

Gene symbol	Primer sequence
GAPDH Forward	TCTCTGCTCCTCCTGTTC
GAPDH Reverse	GTTGACTCCGACCTTCAC
MYEOV Forward	ACTGTTGTGACTGTTGAG
MYEOV Reverse	GTGAGGATGATGATGAGG
GPRC5A Forward	CCAGGATGTTATCGCTATTG
GPRC5A Reverse	TGAAGGAACCACAGAAGG
KRAS Forward	GAGTACAGTGCAATGAGGGAC
KRAS Reverse	CCTGAGCCTGTTTTGTGTCTAC
EGFR Forward	AGGCACGAGTAACAAGCTCAC
EGFR Reverse	ATGAGGACATAACCAGCCACC
SERPINB5 Forward	ACTGAAACTAATCAAGCGGCTC
SERPINB5 Reverse	CTTTGCATACGGTCTCTTCGTAG
EIF4G2 Forward	GGGGTGCTTCTCGTTTCAGT
EIF4G2 Reverse	AGCAGTCTTGGGATAGTGCTG
PDCD4 Forward	GCAAAAAGGCGACTAAGGAAAAA
PDCD4 Reverse	TAAGGGCGTCACTCCCACT

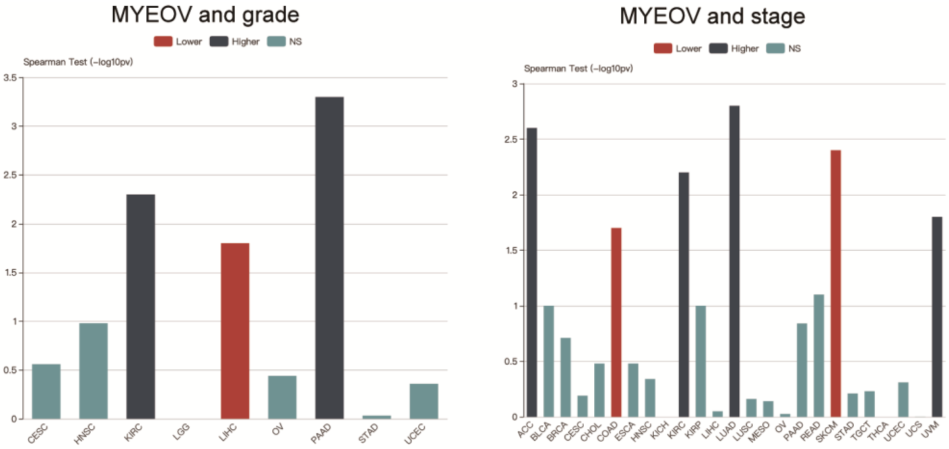

Figure S1. The association of MYEOV with grade and stage in different cancers. MYEOV is associated with higher grade in pancreatic cancer. There is no significant association between MYEOV and higher stage in pancreatic cancer.

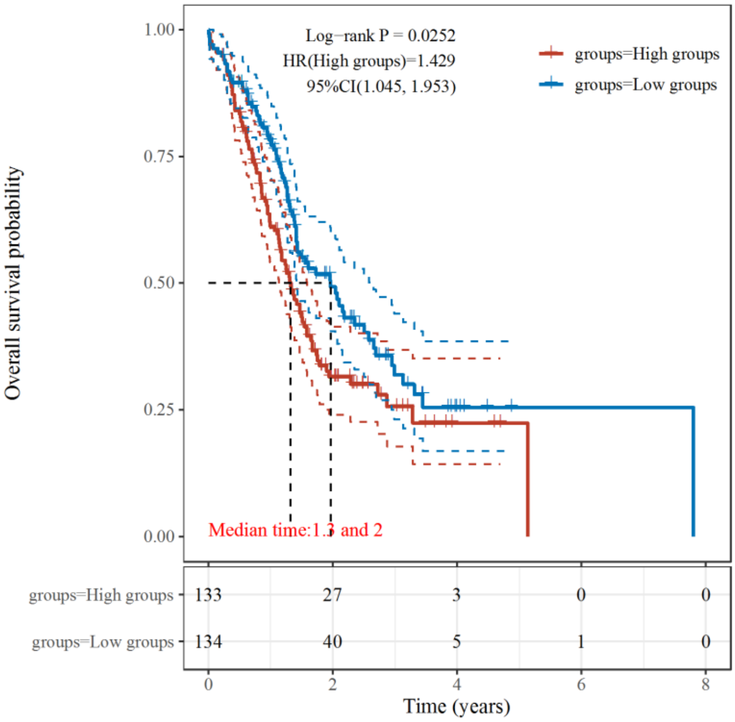

Figure S2. Kaplan-Meier survival analysis by high or low MYEOV expression in the ICGC dataset.

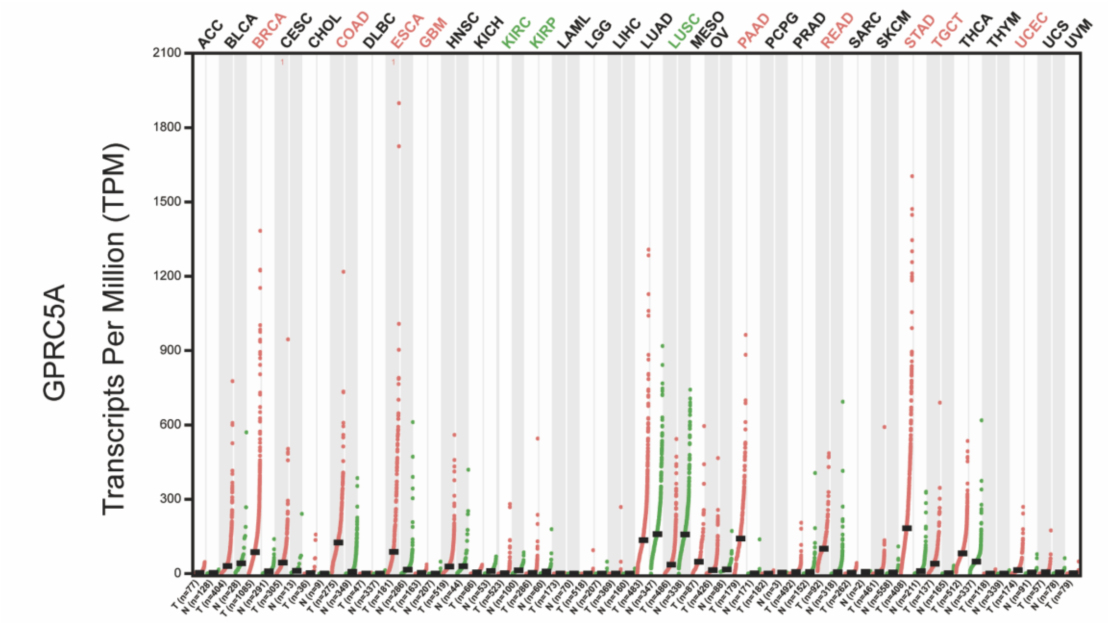

Figure S3. Differential expression analysis of GPRC5A in different cancers.

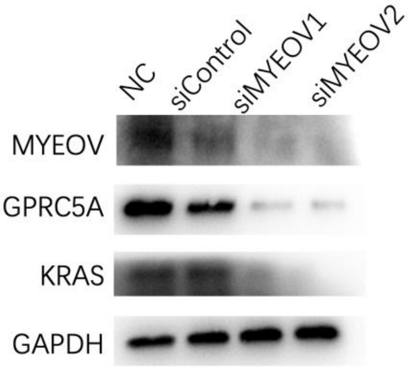

Figure S4. Changes in protein levels of MYEOV, GPRC5A and KRAS after knockdown of MYEOV with siRNAs in CFPAC cells.

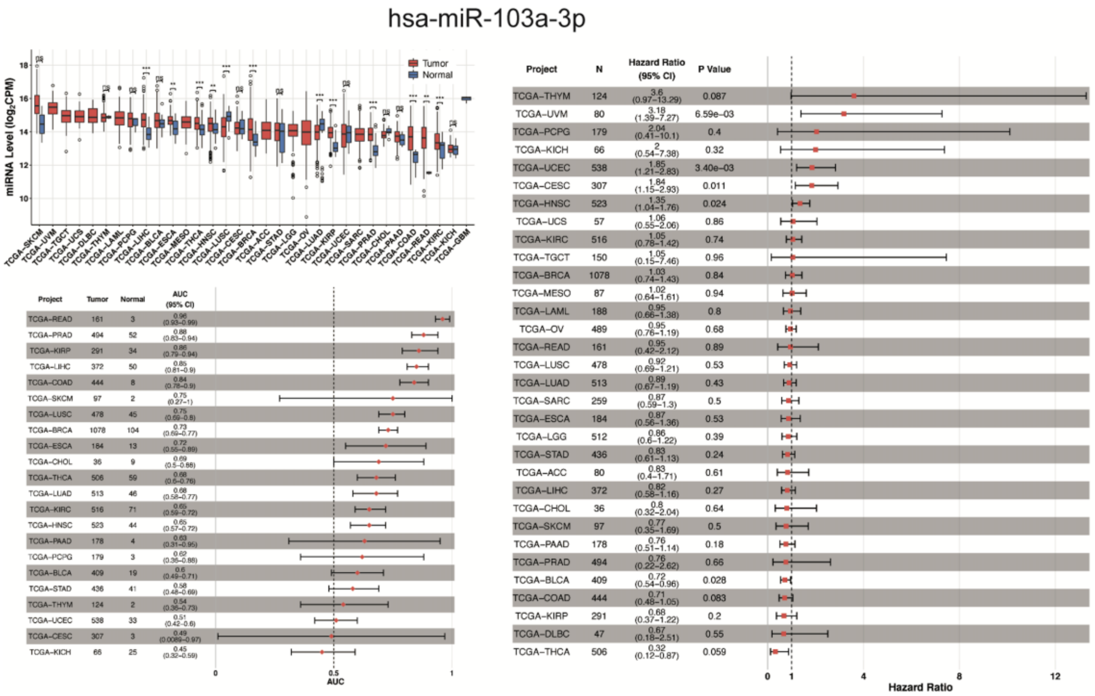

Figure S5. Box plot of differential expression analyses, ROC analyses and survival analyses in different cancers of hsa-miR-103a-3p.

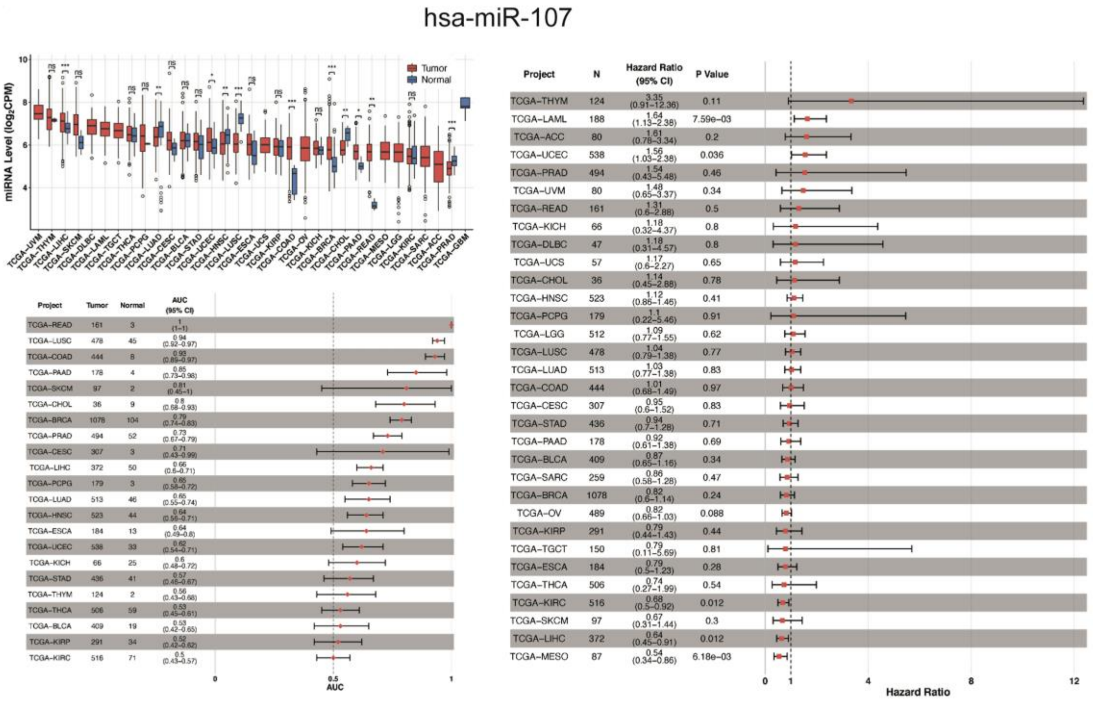

Figure S6. Box plot of differential expression analyses, ROC analyses and survival analyses in different cancers of hsa-miR-107.

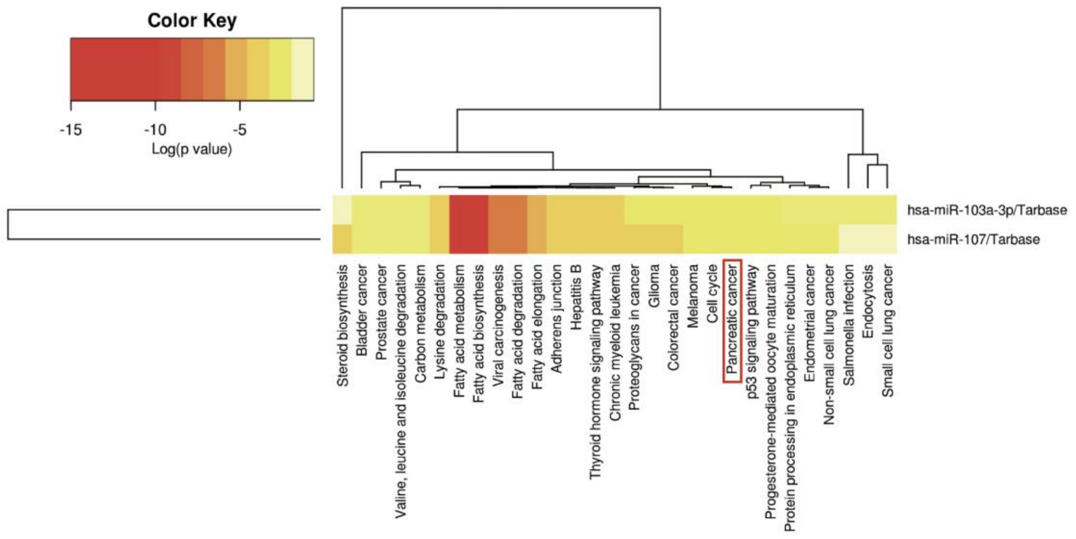

Figure S7. The miRPath database searching results of target gene pathway enrichment analysis for hsa-miR-103a-3p and hsa-miR-107.
